# Favorable prognostic value of SOCS2 and IGF-I in breast cancer

**DOI:** 10.1186/1471-2407-7-136

**Published:** 2007-07-25

**Authors:** Michael C Haffner, Barbara Petridou, Jean Phillipe Peyrat, Françoise Révillion, Elisabeth Müller-Holzner, Günter Daxenbichler, Christian Marth, Wolfgang Doppler

**Affiliations:** 1 Division of Medical Biochemistry, Biocenter, Innsbruck Medical University, Fritz-Pregl-Strasse 3, A-6020 Innsbruck, Austria; 2 Unité GPL, INRA, 78352 Jouy-en-Josas Cedex, France; 3 Centre Oscar Lambret, 59020 Lille Cedex, France; 4 Department of Obstetrics and Gynecology, University of Innsbruck, Anichstrasse 35, A-6020 Innsbruck, Austria

## Abstract

**Background:**

Suppressor of cytokine signaling (SOCS) proteins comprise a protein family, which has initially been described as STAT induced inhibitors of the Jak/Stat pathway. Recent in vivo and in vitro studies suggest that SOCS proteins are also implicated in cancer. The STAT5 induced IGF-I acts as an endocrine and para/autocrine growth and differentiation factor in mammary gland development. Whereas high levels of circulating IGF-I have been associated with increased cancer risk, the role of autocrine acting IGF-I is less clear. The present study is aimed to elucidate the clinicopathological features associated with SOCS1, SOCS2, SOCS3, CIS and IGF-I expression in breast cancer.

**Methods:**

We determined the mRNA expression levels of SOCS1, SOCS2, SOCS3, CIS and IGF-I in 89 primary breast cancers by reverse transcriptase PCR. SOCS2 protein expression was further evaluated by immuno-blot and immunohistochemistry.

**Results:**

SOCS2 expression inversely correlated with histopathological grade and ER positive tumors exhibited higher SOCS2 levels. Patients with high SOCS2 expression lived significantly longer (108.7 vs. 77.7 months; P = 0.015) and high SOCS2 expression proved to be an independent predictor for good prognosis (HR = 0.45, 95% CI 0.23 – 0.91, P = 0.026). In analogy to SOCS2, high IGF-I expression was an independent predictor for good prognosis in the entire patient cohort. In the subgroup of patients with lymph-node negative disease, high IGF-I was a strong predictor for favorable outcome in terms of overall survival and relapse free survival (HR = 0.075, 95% CI 0.014 – 0.388, P = 0.002).

**Conclusion:**

This is the first report on the favorable prognostic value of high SOCS2 expression in primary mammary carcinomas. Furthermore a strong association of high IGF-I expression levels with good prognosis was observed especially in lymph-node negative patients. Our results suggest that high expression of the STAT5 target genes SOCS2 and IGF-I is a feature of differentiated and less malignant tumors.

## Background

SOCS proteins comprise a family of eight members (SOCS1-7 and CIS), which have initially been described as STAT induced STAT inhibitors or more generally as negative regulators of cytokine signaling via the Jak/Stat pathway. Their ability to modulate signal transduction is based on two functional domains, an SH2 domain, which binds to phosphorylated tyrosine residues and a SOCS box, which serves as a recruiting site for ubiquitin ligases [1]. Recent reports, showing the interaction of SOCS proteins with various other signaling molecules like p65, FAK, c-kit, IRS1/2 and vav, indicate a more general role of SOCSs proteins in the maintenance of cellular homeostasis [2-6]. Tumors often exhibit alterations in SOCS protein expression: CpG island methylation of SOCS gene loci, and consequently, inhibition of SOCS expression was observed in a variety of solid tumors and hematological malignancies [7-13]. On the other hand, forced overexpression of SOCS1 and SOCS2 results in diminished transformation and decreased metastatic potential [8,14]. Taken together, these in vivo and in vitro data support the role of SOCS proteins as tumor suppressors. To evaluate the potential significance of SOCS expression in mammary carcinoma for prognosis and its association with clinicopathological characteristics we have investigated the mRNA levels of SOCS1, SOCS2, SOCS3 and CIS in a representative collection of primary breast cancers specimens. Since our study revealed SOCS2 expression as a predictor for prognosis, and growth hormone is a major inducer of SOCS2, we became interested in the expression of the growth hormone regulated gene IGF-I. IGF-I is a growth and differentiation factor, which acts in an endocrine – via the classical hypothalamus/pituitary/liver axis – and in a paracrine/autocrine manner [15]. In the normal mammary gland, IGF-I is required for structural development, promoting ductal growth and differentiation [16]. Whereas epidemiological data indicate that high levels of circulating serum IGF-I are associated with an increased risk for the development of solid tumors including breast cancer, little is known on the role of autocrine produced IGF-I [15,17]. We therefore evaluated the expression levels of locally produced IGF-I in the tumor. Interestingly, high IGF-I mRNA levels were associated with good prognosis, suggesting that secretion of IGF-I by the tumor is a property of a less malignant and more differentiated tumor.

## Methods

### Patients and tumor specimens

Tissue samples from patients treated at the Department of Obstetrics and Gynecology, Innsbruck Medical University, Austria (n = 52, referred to as A), and the Centre Oscar Lambert Anticancer Center of the North of France, Lille (n = 37, referred to as B) for primary breast cancer were included in this retrospective study. Since the initial results obtained in patient sample collection A suggested a prognostic significance of SOCS2 and IGF-I, we further sought to test the variables in another independent patient cohort (collection B) to overcome the potential problem of selection bias and to increase the statistical power. The clinicopathological characteristics of the entire cohort (n = 89) are shown in Table 1. Median age was 62.2 years (range, 35 to 81) and median follow up time was 6.8 years (range, 0.6 to 11.8). During follow up 40 patients relapsed and 37 died. Specimens with a tumor content of more than 90% were snap frozen, ground to powder under liquid nitrogen and stored at -80°C until further analysis. Estrogen and progesterone receptor status was assessed by ligand binding assay as described previously [18].

**Table 1:** Clinical characteristics of patients

Feature		
Total Number	89	
		
Age at diagnosis		
Median	62.2	
Range	35 – 81	
		
Histotype		
Infiltrating ductal carcinoma	60	(67)
Infiltrating lobular carcinoma	10	(11)
Others	19	(21)
		
Histopathological grade		
I	35	(39)
II	24	(27)
III	24	(27)
Not classified	6	(7)
		
Lymph-node status		
Positive	48	(54)
Negative	38	(43)
Unknown	3	(3)
		
Estrogen receptor		
positive	68	(76.4)
negative	21	(23.6)
		
Progesterone receptor		
positive	64	(71.9)
negative	25	(28.1)
		
Follow-up		
Median	6.8	
Range	0.6 – 11.8	

Numbers in parentheses are percentages

### RNA preparation and RT-PCR

RNA was extracted from tissue powder as described previously [19]. Integrity of prepared RNA was evaluated by determination of ethidiumbromide-stained 18S and 28S-ribosomal RNA bands in an agarose gel. 0.5 μg of total RNA were reverse transcribed with Superscript III (Invitrogen, Carlsbad, CA, USA) according to manufacturer's instructions. Primer and Taq-Man probe oligonucleotide sequences for SOCS1, SOCS2, SOCS3, CIS, IGF-I and TATA box-binding protein are shown in Table 2. All PCR reactions were carried out on an ABI Prism thermocycler (PerkinElmer, Inc., Wellesley, MA, USA) at 95°C for 10 min followed by 40 cycles of 95°C for 15 s and 60°C for 30 s in a 25 μl volume containing 1× BioTherm Star™PCR Buffer (GeneCraft, Lüdinghausen, Germany), 5 mM MgCl_2_, 1U Taq-Polymerase BioThermStar™ (GeneCraft), 300 μM dATP, 300 μM dGTP, 300 μM dCTP, 300 μM dTTP, 200 nM forward primer, 200 nM reverse primer, 100 nM of TaqMan probe, 300 nM reference dye (Stratagene, La Jolla, CA), and 2 μl of cDNA. To control for variations in RNA quality and quantity, expression of the gene of interest was normalized to the expression of TATA box binding protein (TBP) [20]. Each reaction included a standard-control sample and a non template control. To test for amplification efficiency, series dilutions of the control samples were analyzed. Representative amplification plots are shown [see Additional file 1]. Relative mRNA expression levels were calculated according to the formula: 2^-ΔΔCT^, where ΔCT_sample _was defined as CT_gene of interest _– CT_TBP _and ΔΔCT as ΔCT_sample _– ΔCT_normalization sample_.

**Table 2:** Primers and Probes for real-time PCR

Gene Symbol		Sequence
SOCS1	Forward	5'-TTTTCGCCCTTAGCGTGAAG-3'
	Reverse	5'-CATCCAGGTGAAAGCGGC-3'
	Taqman probe	5'FAM-CCTCGGGACCCACGAGCATCC-3'TAM
		
SOSC2	Forward	5'-CAGATGTGCAAGGATAAGCGG-3'
	Reverse	5'-CAGATAAAGGTGAACAGTGCCG-3'
	Taqman probe	5'FAM-CAGGTCCAGAAGCCCCCCGG-3'TAM
		
SOCS3	Forward	5'-TGATCCGCGACAGCTCG-3'
	Reverse	5'-TCCCAGACTGGGTCTTGACG-3'
	Taqman probe	5'FAM-CCAGCGCCACTTCTTCACGCTCA-3'TAM
		
CIS	Forward	5'-TCCAACTGCTTGTCCAGGC-3'
	Reverse	5'-GTGCTGCACAAGGCTGACC-3'
	Taqman probe	5'FAM-ACGCATCCTGGCCTTTCCGGA-3'TAM
		
TBP	Forward	5'-CACGAACCACGGCACTGATT-3'
	Reverse	5'-TTTTCTTGCTGCCAGTCTGGAC-3'
	Taqman probe	5'FAM-TCTTCACTCTTGGCTCCTGTGCACA-3'TAM
		
IGF-I	Forward	5'-TCAGCTCGCTCTGTCCGTG-3'
	Reverse	5'-TGACTCCCTCTACTTGCGTT-3'
	Taqman probe	5'FAM-TGCCCAAGACCCAGAAGGAAGTACATTTG-3'TAM

### Immunohistochemistry

COS 7 monkey kidney cells were grown in DMEM containing 10% FCS at 37°C in 5% CO_2_/95% air. Cells were transiently transfected with a myc tagged SOCS2 expression plasmid [21] using TransFast™ transfection reagent (Promega, Madison, WI). Thirty six hours after transfection cells were trypsinized and centrifuged at 1000 g for 10 min. The cell pellet was then resuspended in a 4% low-melting agarose (United States Biochemical, Cleveland, Ohio) PBS solution. After hardening of the agarose, the gel pellet was incubated overnight in 4% paraformaldehyde for 12 h, paraffin embedded and processed for immunohistochemistry with an anti-myc or anti-SOCS2 antibody to assess the specificity of the antibody against SOCS2.

Immunohistochemistry was performed a described previously [22]. Primary anti-SOCS2 antibody (FA1016, Fusion Antibodies Ltd, Belfast, Ireland) was applied at 1:50 dilution for 30 min at room temperature. Eight cases for which both paraffin embedded and frozen tissue were available were investigated.

### Western Blot

Whole cell extracts from pulverized tumour and normal adjacent tissue were prepared as described previously [18]. Membranes were then probed with an anti-SOCS2 rabbit polyclonal antibody (FA1016, Fusion Antibodies Ltd, Belfast, Ireland) at 1:500, mouse monoclonal anti-actin (C-2, Santa Cruz Biotechnology, Santa Cruz, CA) 1:200 and further incubated in 1:5000 anti-rabbit IR 800 secondary antibody (Rockland, PA) and 1:5000 anti-mouse IR 680 secondary antibody (Molecular Probes, Leiden, Netherlands), respectively. Immunoreactive bands were detected using a LICOR Odyssey Infrared Imager (LI-COR, Biosciences, Lincoln, NB). To test for antibody specificity, lysates from COS7 cells transiently transfected with a myc-tagged SOCS2 plasmid were probed with an anti-c-myc antibody (9E10, Santa Cruz Biotechnology, Santa Cruz) and anti-SOCS2 antibodies. Both antibodies recognized the recombinant protein and showed an immunoreactive band at the estimated molecular weight.

### Statistical analysis

Correlations between parameters were assessed according to the Spearman nonparametric test. Differences in the distributions of SOCS2 and IGF-I between tumor and normal adjacent tissue were compared using Mann-Whitney test. The Kruskal-Wallis one-way ANOVA was used to test for differences in SOCS and IGF expression levels between histopathological grade groups. Overall survival and relapse free survival between the mRNA expression categories were compared using Kaplan-Meier plots and the log-rank test. The median was determined as an optimal cut-off value to separate the low and high expression categories. A COX proportional hazards regression model was used to estimate the hazard ratio (HR) associated with SOCS1, SOCS2, IGF-I expression. The final multivariate model included lymph node-status, estrogen and progesterone receptor status and histopathological grade. All statistical analysis were performed using SPSS 11.0 for Mac OS (SPSS, Inc., Chicago, IL). A P-value < 0.05 was considered statistically significant.

## Results

### SOCS and IGF-I mRNA and clinicopathological features

The mRNA content of SOCS1, SOCS2 and IGF-I was assessed in 89 primary breast cancer samples, and of SOCS3 and CIS in 64 samples. The patient characteristics are shown in Table 1. In all breast tumors transcripts of the investigated genes were detectable and quantifiable.

#### SOCS1

SOCS1 expression was negatively associated with ER and PR levels (r = -0.224, P = 0.037; and r = -0.239, P = 0.026, respectively). The group of patients with low SOCS1 expression exhibited longer overall survival (100.3 versus 84.7 months). This association of low SOCS1 expression and good prognosis reached border-line statistical significance (P = 0.07) and is in line with recent findings from our group, showing that activation of STAT1 is an indicator for favorable outcomes in mammary carcinoma, since SOCS1 is a negative regulator of STAT1 activation [18].

#### SOCS2

**Figure 1:**
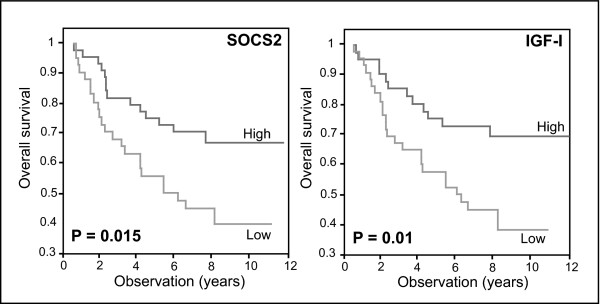
Kaplan-Meier curve assessment of risk of death in a cohort of 89 patients with invasive breast cancer. Overall survival is shown for patients with high and low SOCS2 and IGF-I expression, respectively. The median was taken as a cut-off. P values were determined using the log-rank test.

A statistical highly significant correlation was observed between SOCS2 expression and pathological grade (r = -0.306, P = 0.005). There, tumors with low histopathological grade had higher SOCS2 mRNA content. This is in agreement with recent findings showing that high SOCS2 protein expression is associated with lower pathological grade and lower cell proliferation indices [23]. Furthermore, tumors with high SOCS2 content tended to be estrogen (r = 0.257, P = 0.015) and progesterone receptor positive (r = 0.246, P = 0.02) and significantly higher SOCS2 levels were observed in the subgroup of ER positive tumors (P = 0.04) indicating a general tendency for more differentiated tumors to have high SOCS2 expression. It was of particular interest, that patients with high SOCS2 expression lived significantly longer (108.7 vs. 77.7 months; P = 0.015; Figure 1). The difference in disease free survival was in the same direction but did not reach statistical significance (91.9 versus 74.3 months; P = 0.22). After adjusting for tumor grade, lymph node, ER and PR status high SOCS2 expression remained an independent predictor for good prognosis (Table 3). This is the first evidence for a prognostic relevance of SOCS expression in breast cancer.

**Table 3:** Multivariate Cox regression analysis for overall survival

Variable		Hazard ratio	95% confidence interval	P
SOCS2				
	less than median	1.00		0.026
	greater than median	0.453	0.23 – 0.91	
IGF-I				
	less than median	1.00		0.017
	greater than median	0.414	0.23 – 0.85	

#### SOCS3 & CIS

CIS correlated with estrogen receptor (r = 0.273, P = 0.029), and progesterone receptor status (r = 0.272, P = 0.03). For neither CIS nor SOCS3 there was a statistically significant association between mRNA expression levels and overall or relapse free survival. We observe a positive correlation of CIS and SOCS2 mRNA levels (r = 0.25, P = 0.047). This is in agreement with the notion that both CIS and SOCS2 have been described as STAT5 target genes [24].

#### IGF-I

**Figure 2:**
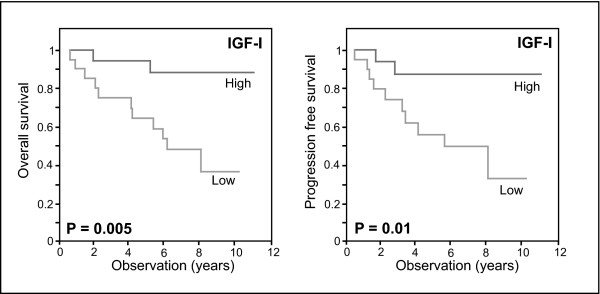
Kaplan-Meier curve assessment of risk of death and risk of disease recurrence in 37 patients with lymph-node negative disease. Overall survival and relapse free survival (progression free survival) is shown for patients with high (17 pts.) and low (20 pts.) IGF-I expression.

We included IGF-I as another STAT5 regulated gene in our study. Tumors with high IGF-I expression tended to be ER and PR positive (r = 0.24, P = 0.028; r = 0.27, P = 0.011). Furthermore, a strong positive correlation between high SOCS2 and high IGF-I expression was observed (r = 0.4, P < 0.001). In analogy to SOCS2, IGF-I levels above the median were associated with significantly longer overall survival (76.3 versus 110.5 months; P = 0.01; Figure 1), and showed a borderline significant association to longer disease free survival (70.9 versus 96.1 months; P = 0.082). In a multivariate COX regression model we were able to show the prognostic relevance of high IGF-I expression (Table 3). This is in line with previously reported data [25]. Subgroup analysis in 30 patients who had both, high SOCS2 and high IGF-I expression did only show a minor advantage for the combination of the two markers in terms of predicting overall survival (118.5 versus 79.55 months; P = 0.005). When we restricted our analysis to lymph-node negative patients, who are known to have better prognosis than lymph-node positive patients [26], we observed an even more pronounced difference in overall survival and relapse-free survival (Figure 2): There, low IGF-I expression defined a subgroup of patients who were at very high risk for recurrence. High IGF-I expression, conversely was associated with a significantly decreased risk for recurrence (HR = 0.075, 95% CI 0.014 – 0.388, P = 0.002).

### SOCS2 and IGF-I in tumor and normal adjacent tissue

**Figure 3:**
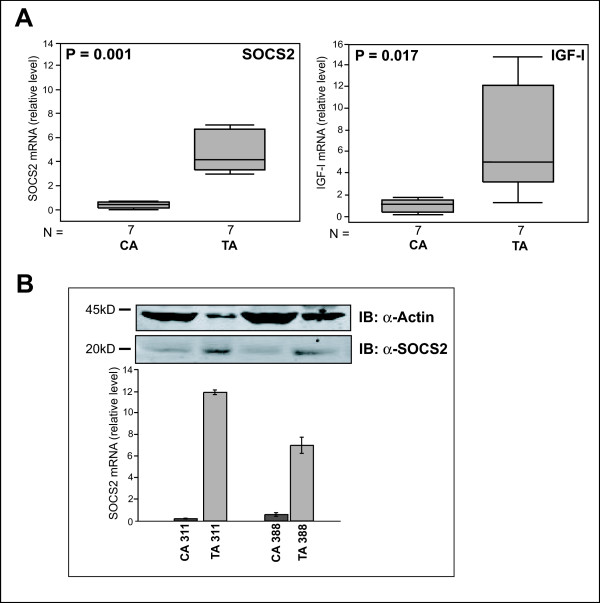
Comparison of SOCS2 and IGF-I expression. (A) Box blots with data from a set of seven tumors comparing the relative mRNA expression levels in tumor (CA) and normal adjacent tissue (TA). (B) SOCS2 protein and corresponding SOCS2 mRNA expression in normal and cancerous tissue of two tumor specimens. SOCS2 protein expression was assessed by immuno-blot with an antibody directed against SOCS2. Actin was used as a loading control.

**Figure 4:**
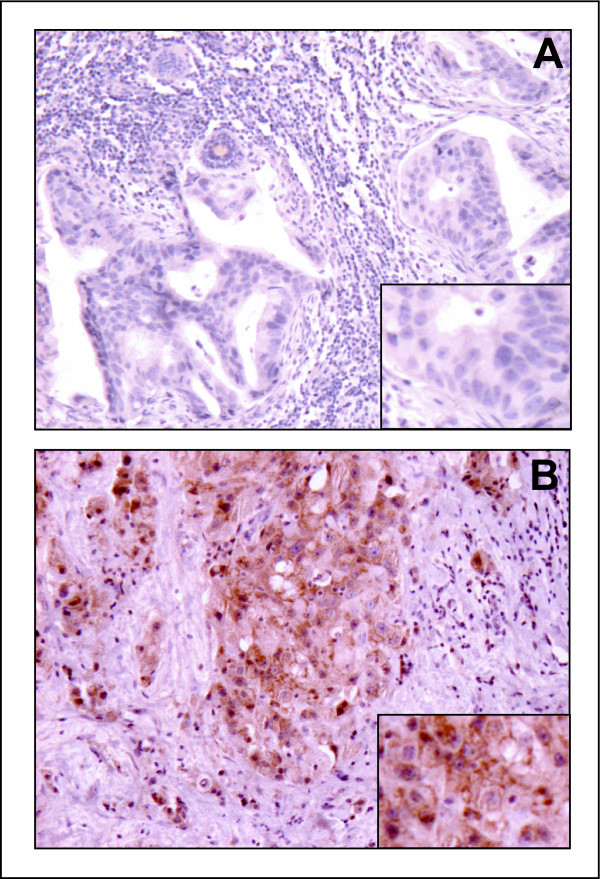
Detection of SOCS2 in primary mammary carcinoma samples by immunohistochemistry. Representative immunohistochemical stainings of samples with low SOCS2 (A) and high SOCS2 (B) mRNA expression are shown. Note a strong cytoplasmatic epithelial staining for SOCS2 in tumors with high SOCS2 mRNA levels (B).

We further compared the mRNA expression of SOCS2 and IGF-I in primary breast carcinomas (CA) and the corresponding normal adjacent tissue (TA) in a set of seven tumors. The mean SOCS2 and IGF-I expression levels were 9.4 and 8.4 fold higher, respectively, in TA than in CA (Figure 3A). SOCS2 was significantly correlated with IGF-I expression (r = 0.736, P = 0.003). SOCS2 mRNA data were further corroborated by Western blot analysis (Figure 3B) with an antibody evaluated for its specificity by experiments with cells expressing recombinant SOCS2 protein (see Methods section). The same antibody was also found to be suitable for immunohistochemistry. There, in primary mammary carcinoma immunohistochemical staining of SOCS2 correlated with SOCS2 mRNA expression. In tumors with high SOCS2 mRNA levels, epithelial tumor cells showed cytoplasmatic staining for SOCS2 (Figure 4). In normal breast tissue, ducts, and to a lesser extent, stroma cells exhibited staining for SOCS2 (not shown). Although SOCS2 protein content was not measured in all samples of our tumor collection, the high correlation between SOCS2 protein and mRNA expression in 14 samples investigated by westernblot analysis and in 8 samples by immunohistochemistry suggests that SOCS2 mRNA levels, as determined by RT-PCR are a suitable indicator for how much SOCS2 protein is expressed.

## Discussion

Several recent reports have investigated the role of SOCS proteins in the oncogenesis of various solid tumors and hematological malignancies [7-13] indicating a central role of SOCS proteins in the regulation of cellular growth and differentiation. SOCS promoter methylation and consequent gene silencing has been observed in hepatocellular carcinoma, lung cancer, head and neck cancer, multiple myeloma and AML [9-13]. Conversely, restoration of SOCS protein expression resulted in induction of apoptosis and suppression of growth. These findings suggest an antioncogenic role of SOCS proteins.

However, little is known on the prognostic significance and the association of SOCS expression with clinicopathological features. In this study, we therefore sought to elucidate the relationship between pathological and clinical parameters and the expression of the SOCS family members SOCS1, SOCS2, SOCS3, and CIS in patients with breast cancer by RT-PCR.

Our results showed that high SOCS2 expression is associated with increased overall survival and after adjusting for age, lymph-node status, estrogen receptor status and tumor grade, high SOCS2 expression remained an independent predictor for good prognosis (Table 3). Farabegoli et al. recently provided first evidence that high SOCS2 protein expression is associated with lower pathological grade and lower cell proliferation indices [23]. Together with our findings of highly significant negative correlation between SOCS2 expression and histopathological grade, positive correlation with steroid receptors, which are markers of differentiation, and the higher expression in normal tumor adjacent tissue as compared to carcinoma tissue, these results indicate that SOCS2 is a marker for differentiated tumors. Similar to our observations in breast cancer low SOCS2 expression in prostate cancer is associated with an increased incidence of metastasis and SOCS2 mRNA levels decrease during prostate cancer progression [27]. Furthermore, SOCS2 expression was found to be down-regulated in pulmonary adenocarcinoma [28]. The important role of SOCS2 as a regulator of growth hormone signaling raises the intriguing question, whether in mammary carcinoma, SOCS2 serves as an essential regulator of cellular growth and tissue homeostasis and its expression is required for the maintenance of a more differentiated and less malignant phenotype. However, Raccurt et al. described an increased expression in cancerous ducts and reactive stroma as compared to normal breast tissues by using in situ hybridization [29]. This result is not in agreement with our findings obtained by RT-PCR, immuno-blotting and immunohistochemistry and might be due to the differences in the detection methods.

The fact that SOCS2 has been described to be induced by and to modulate the GH receptor/Jak/STAT pathway prompted us to further investigate the expression of another downstream target of the GH receptor, IGF-I [30,31]. Similarly to our observations with SOCS2, high IGF-I mRNA expression proved to be a predictor for good prognosis (Figure 1 &2), and normal adjacent tissue was characterized by increased IGF-I mRNA levels (Figure 3). Our study confirms the results from a previous report from a large sample collection in which patients with high IGF-I expression tended to have a favorable prognosis [25].

Mammary epithelial cell specific overexpression of IGF-I which mimics autocrine IGF-I, increased the incidence of mammary carcinomas in mice [32], whereas liver-specific deletion of IGF-I, eliminating serum IGF-I, results in reduced tumor development [33]. Several large epidemiological studies highlighted this association also in humans, revealing an increased risk for breast cancer especially in women with elevated serum levels of IGF-I before the age of 50 [34]. Compiled evidence suggests that high IGF-I renders cells susceptible to transformation by secondary events and thereby contributes to tumor progression [15]. We observed an increased expression of IGF-I in normal tumor adjacent tissue as compared to cancerous tissue. This is in line with a previous reports comparing IGF-I expression in large collections of normal mammary glands and breast cancer specimens [25,35]. Differences in IGF-I expression levels between normal and cancerous tissue could partly be explained by differences in the prevailing cell types. Several reports showed that IGF-I expression is restricted to stroma cells in the mammary gland [36]. High stroma content, frequently found in highly differentiated tumors would therefore result in higher IGF-I levels, which could explain the positive prognostic value of high IGF-I expression. However, MCF7 and T47D mammary carcinoma cells showed expression of IGF-I (Haffner MC, Doppler W, unpublished observation) indicating that also epithelia cells are capable of producing IGF-I.

Interestingly, we observed that high IGF-I expression is a very potent predictor for good prognosis in patients with lymph-node negative disease (Figure 2). Histological status of the axillary lymph-nodes represents an important prognostic factor. Patients with even a single lymph-node metastasis have a poorer outcome than those with negative nodes and decision on systemic chemotherapy is largely based on lymph-node status [26]. Risk stratification in patients without lymph-node involvement is difficult. Hence markers predicting the outcome of patients in this subgroup are needed. Gene-expression profiling based on microarray analysis of 70 genes is a powerful predictor for disease outcomes in patients with lymph-node negative disease. [37] We demonstrate here in a limited number of patients that expression levels of a single gene (IGF-I) can be used to independently predict the risk of disease recurrence and death in lymph-negative patients. Further studies evaluating this marker in a larger cohort of patients are needed to determine its prognostic and predictive potential.

Our results point to the hypothesis that both IGF-I and SOCS2 expression in breast cancer is controlled by similar mechanisms. A possible common signaling intermediate could be STAT5, since both IGF-I and SOCS2 have been described as STAT5 target genes [38,39]. STAT5 is essential for mammary gland development and lactogenesis [40,41], and alterations and repression in the signaling cascade could result in less differentiated and more malignant tumors. This is in line with recent findings showing that breast lesions with aberrantly proliferating cells like atypical ductal hyperplasia, ductal carcinoma in situ and invasive carcinomas are characterized by a reduction or absence of STAT5a expression [42]. Activation of STAT5, as determined by tyrosine phosphorylation, decreases during metastatic progression of breast cancer, and in a large patient cohort, activated STAT5 emerged as an independent prognostic marker for good prognosis in women with breast cancer [43]. Furthermore, STAT5 expression levels in the primary tumor as determined by immunohistochemisty was associated with better response to endocrine therapy suggesting a possible crosstalk between ER and STAT5, which could be mediated by SOCS2 [44]. Interestingly, CIS, another known STAT5 target gene, was strongly correlated with SOCS2 and IGF-I, but did not prove to be a prognostic marker.

Whereas SOCS2 was initially described as a negative regulator of GH signaling [30], recent reports suggest that depending on the expression level, SOCS2 can act as either an enhancer or suppressor of this signaling pathway [45,46]. SOCS2 knock out mice show only modest increase in STAT5 phosphorylation [39] and overexpression of SOCS2 even results in an enhancement of GH signaling in vivo and in vitro [46]. Therefore, high STAT5 activation in the tumor is compatible with higher SOCS2 levels. Interestingly, GH-transgenic mice lacking one copy of SOCS2 show a high incidence in aberrant lesions in the colon indicating that small variations of SOCS2 expression levels can have profound implications on cell proliferation and eventually tumor growth [47].

In a comprehensive study Kate Sutherland et al. evaluated promoter CpG island methylation and loss of heterozygosity (LOH) of the SOCS1, SOCS2 and SOCS3 genes in ovarian and breast cancer [8]. In 48 primary breast cancer samples no methylation was observed and only 13% of tumors showed LOH in the SOCS2 locus. Similar, unpublished results from our group show no significant CpG island methylation in SOCS2 locus in a limited number of breast cancer samples (Haffner MC and Auer D, unpublished data). In conclusion, the current data do not support methylation as a major factor contributing to differences in SOCS2 expression levels in primary breast cancers.

Our observation of higher SOCS2 expression levels in ER positive tumors, raises the hypothesis that functional ER signaling contributes to enhanced SOCS2 transcription. This is in accordance with a recent report showing selective upregulation of SOCS2 expression by estrogen in vitro [48]. In addition, mice carrying a targeted deletion of the estrogen receptor failed to show an increased SOCS2 expression upon estrogen stimulation [49]. It still remains to be shown whether high SOCS2 expression per se is causative for the differences in tumor differentiation and prolonged overall survival. Preliminary results from MCF7 mammary carcinoma cells show that overexpression of SOCS2 results in a significant reduction of anchorage independent growth, which supports the hypothesis of SOCS2 as an antioncogene [8]. Whether the favorable prognostic characteristics of ER positive tumors can be attributed to some extent to higher SOCS2 expression needs to be further clarified.

## Conclusion

This is the first report on the prognostic significance of SOCS2 expression in breast cancer. We further provide new evidence that high expression of IGF-I is associated with favorable prognosis especially in patients with lymph-node negative disease. Taken together our findings contribute to a better understanding of the function of SOCS proteins and IGF-I expression in breast cancer. Furthermore, if confirmed in larger studies, determination of SOCS2 and IGF-I expression levels could be used in clinical decision making for patients with breast cancer.

## Abbreviations

CIS, cytokine-inducible SH2 containing protein; ER, estrogen receptor; GH, growth hormone; HR, hazard ratio; IGF-I, insulin-like growth factor 1; LN, lymph node; PR, progesterone receptor; SOCS, suppressors of cytokine signaling; STAT, signal transducer and activator of transcription.

## Competing interests

The author(s) declare that they have no competing interests.

## Authors' contributions

MCH conceived the study, performed experiments and wrote the paper. BP participated in RT-PCR analysis and study design. JPP and FR contributed patients' samples and clinical data. EM-H carried out the pathological studies. GD and CM contributed to study design and provided samples. WD conceived and coordinated the study and wrote the manuscript. All authors read and approved the final manuscript.

## Pre-publication history

The pre-publication history for this paper can be accessed here:



## Supplementary Material

Additional file 1 Representative RT-PCR amplification plots. Representative real-time PCR amplification plots for SOCS2, IGF-I and TBP in primary mammary carcinoma samples. Each reaction included a standard-control sample and a non- template-control together with up to 45 patient samples. All reactions were performed in duplicates. Detected Ct-values ranged from 22.48 to 30.81 for SOCS2, 23.8 to 31.87 for IGF-I and 22.0 to 26.72 for TBP. Click here for file
